# *Bupleurum* in Treatment of Depression Disorder: A Comprehensive Review

**DOI:** 10.3390/ph17040512

**Published:** 2024-04-16

**Authors:** Shuzhen Ran, Rui Peng, Qingwan Guo, Jinshuai Cui, Gang Chen, Ziying Wang

**Affiliations:** Interdisciplinary Institute for Personalized Medicine in Brain Disorders, School of Traditional Chinese Medicine, Jinan University, Guangzhou 510632, China; ranshuzhen@stu2021.jnu.edu.cn (S.R.); pengrui@stu2021.jnu.edu.cn (R.P.); guoqingwan2255@163.com (Q.G.); zyxzcjs@163.com (J.C.)

**Keywords:** *Bupleurum*, active ingredients, antidepressant, depression, Chinese traditional formula

## Abstract

The incidence of depression has been steadily rising in recent years, making it one of the most prevalent mental illnesses. As the pursuit of novel antidepressant drugs captivates the pharmaceutical field, the therapeutic efficacy of Traditional Chinese Medicine (TCM) has been widely explored. Chaihu (*Bupleurum*) has been traditionally used for liver conditions such as hepatitis, liver inflammation, liver fibrosis, and liver cancer. It is believed to have hepatoprotective effects, promoting liver cell regeneration and protecting against liver damage. In addition, *Bupleurum* has also been used as a Jie Yu (depression-relieving) medicine in China, Japan, Republic of Korea, and other Asian countries for centuries. This review article aims to summarize the research conducted on the antidepressant properties and mechanisms of *Bupleurum*, as well as discuss the potential of TCM formulas containing *Bupleurum*. This review highlights various antidepressant ingredients isolated from *Bupleurum*, including saikosaponin A, saikosaponin D, rutin, puerarin, and quercetin, each with distinct mechanisms of action. Additionally, Chinese herb prescriptions and extracts containing *Bupleurum*, such as Chaihu Shugansan, Xiaoyaosan, and Sinisan, are also included due to their demonstrated antidepressant effects. This review reveals that these *Bupleurum* compounds exhibit antidepressant effects through the regulation of neurotransmitter mechanisms (such as 5-HT and DA), the NMDA (N-methyl-D-aspartate) system, brain-derived neurotrophic factor (BDNF), and other intracellular signaling pathways. Collectively, this comprehensive review provides insights into the multiple applications of *Bupleurum* in the treatment of depression and highlights its potential as an alternative or complementary approach to traditional therapies. However, it is essential to consider the potential adverse effects and clinical restrictions of *Bupleurum* despite its promising potential. Further research is needed to elucidate its specific mechanisms of action and evaluate its effectiveness in human subjects.

## 1. Introduction

Depression is a prevalent and persistent mental disorder characterized by pervasive and persistent low mood, interest, and motivation, and even suicidal tendencies [1]. It imposes a significant socioeconomic burden, including increased healthcare spending and elevated suicide rates [2,3]. Despite numerous hypotheses regarding its pathogenesis, the precise etiology remains unclear, and accurate diagnostic and treatment modalities are lacking [4]. An important consideration in the selection of an antidepressant is its safety and tolerability. Selective serotonin reuptake inhibitors (SSRIs), serotonin–norepinephrine reuptake inhibitors (SNRIs), and tricyclic antidepressants (TCAs) are the three most commonly used classes of antidepressants in clinical practice. The common cardiovascular side-effects [5,6,7,8,9], gastrointestinal side-effects [10,11], hepatotoxicity [12,13], and sexual dysfunction [14,15,16] are reported. A considerable number of patients had delayed action time after taking the drug, showing resistance to the treatment [17,18]. Rapid antidepressant N-methyl-D-aspartate receptor (NMDAR) antagonists such as ketamine have concerns about addiction, safety, and controllability [19,20,21]. *Bupleurum* has been used as a Jie Yu (depression-relieving) medicine [22] in China, Japan, Republic of Korea, and other Asian countries for centuries. However, there is a lack of further research and development on the antidepressant active substances in it, which may provide new ideas for the improvement in antidepressant drugs.

*Bupleurum radix*, also referred to as Chai Hu in China, is derived from the dried root of *Bupleurum Chinese DC*. Or *Bupleurum scorzonerifolium Willd.* The Chinese pharmacopoeia describes Chai Hu as possessing a pungent, bitter, and slightly cold nature. It is said to have the functions of reducing fever, relieving liver stagnation, and elevating Yang Qi. More than 250 compounds have been isolated and identified from *Bupleurum* in recent years [23]. The main ones are triterpene saponins, essential oils, flavonoids, lignans, and polysaccharides [24,25,26]. Clinically, Chai Hu is often used to treat conditions such as colds, fever, chest and flank pain, irregular menstruation, uterine prolapse, and anal prolapse. In recent years, research on *Bupleurum* has primarily focused on its biological activities, including its anti-inflammatory [27,28], anticancer [29], antipyretic [30], antiviral [31], liver protection [32], and immune regulation properties [33]. Recent investigations have revealed that *Bupleurum* can improve depression-like behavior in mice and display antidepressant effects [34,35]. The meta-analysis of randomized controlled trials of the *bupleurum* Chinese herbal formula in managing depression revealed that the *bupleurum* Chinese formula alone or given as integrative medicine with antidepressants reduced depression severity [36,37]. In this review, we provide a comprehensive summary of the pharmacological components of *Bupleurum* and the latest empirical evidence for its antidepressant effects. Additionally, we also list the antidepressant mechanisms of its related traditional Chinese medicine (TCM) prescriptions, which can provide new insights for the development of new clinical antidepressants with minimal side-effects.

## 2. The Pathological Underpinnings of Depression

**Figure 1:**
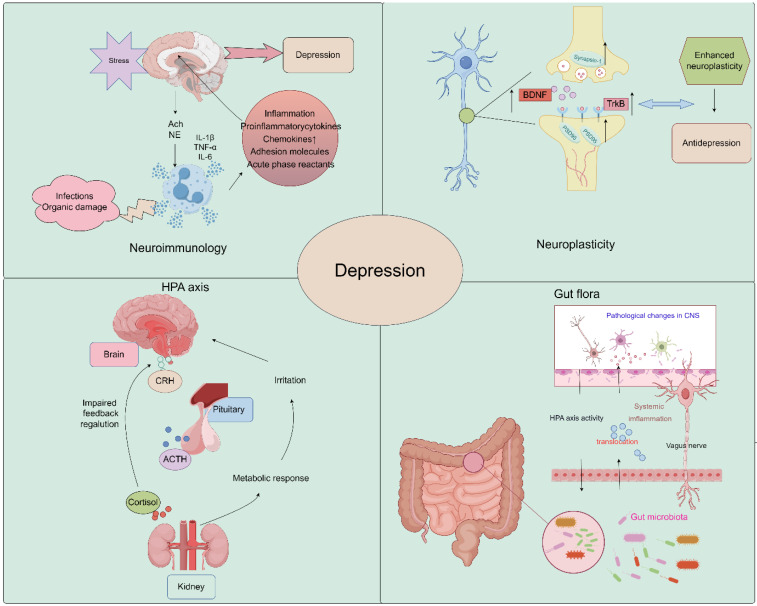
Schematic image of pathological underpinnings of depression (by Figdraw).

The pathogenesis of depression remains a complex and multifaceted phenomenon, with numerous theories proposed to explain its underlying mechanisms. The widely accepted theories of the pathogenesis of depression include neuroimmunity, neuroplasticity, neuroendocrine factors, the monoamine hypothesis, and gut microbiology theory. These theories offer diverse perspectives on the biological, genetic, and environmental factors that contribute to the onset and progression of depression. By exploring the interplay between these theories, researchers may gain a more comprehensive understanding of the pathogenesis of depression and develop more effective diagnostic and therapeutic strategies (Figure 1).

### 2.1. Neuroplasticity

Growth and adaptation at the neuronal level, referred to as neuroplasticity, may be altered by inflammation and hypothalamus–pituitary–adrenal (HPA) axis dysfunction caused by environmental stress [38,39]. A major depressive disorder is associated with reduced neurogenesis processes controlled by regulatory proteins, such as brain-derived neurotrophic factor (BDNF) [40,41]. Studies have demonstrated that Saikosaponin A, a triterpenoid saponin found in bupleurum, exerts significant upregulatory effects on the expression of p-CREB and BDNF, while it downregulates the expression of Bax and Caspase-3. It promotes the expression of Bcl-2, thereby preventing neuronal apoptosis and ameliorating depression-like behavior following cerebral ischemia [35]. Liu et al. demonstrated that Chaihu-Shugan-San, a traditional Chinese medicine formulation primarily composed of Chaihu, exerts antidepressant effects by enhancing synaptic plasticity in the hippocampus [42].

### 2.2. Neuroimmunology

In recent years, several new psychiatric studies have hypothesized that inflammatory processes are involved in the pathogenesis of major depressive disorder. A recent study showed that Interleukin-6 (IL-6) was downregulated in the CA1 hippocampus in two animal models of depression, chronic unpredictable mild stress (CUMS) and lipopolysaccharide (LPS), and was upregulated by antidepressants. Conversely, IL-6 downregulation exacerbated neuronal abnormalities within the CA1 region and promoted the development of a depressive phenotype in rats [43]. In addition, Kynurenine (Kyn) is a pro-inflammatory metabolite in neuroimmune signaling networks that mediate depression-like behaviors. Intraperitoneal injection of Kyn activated the Nod-like receptor protein 2 (NLPR2) inflammasome in mouse hippocampal astrocytes, and NLRP2 knockdown in astrocytes abolished Kyn-induced depression-like behavior in mice [44]. Therefore, dysregulated innate immune responses can increase stress sensitivity and promote the development of depressive behaviors. These findings highlight the potential role of inflammatory processes in the etiology of depression and provide new insights into the development of novel therapeutic strategies for this debilitating condition [45]. *Bupleurum* contains a variety of flavonoids, which have a good effect on improving neuroinflammation and achieving an antidepressant effect [46,47,48].

### 2.3. Neuroendocrine Factors

The HPA axis represents a cardinal neuroendocrine stress response system that maintains homeostasis by adapting the organism to fluctuating demands. Stress and acute challenges have long been recognized as potential risk factors for depression, which frequently co-occur with depressive episodes. Depressive symptoms may therefore be modulated by aberrations in the HPA axis in response to stress [49,50]. It has been shown that Saikosaponin D, one of the active components of *Bupleurum*, can mediate the depressive effect on CUMS model mice by enhancing the function of the HPA axis [51].

### 2.4. Monoamine Hypothesis

During the mid-20th century, it was observed that reserpine, an antihypertensive medication, elicited major depressive disorder symptoms and decreased levels of monoamine neurotransmitters. This finding sheds light on reduced levels of serotonin (5-HT), norepinephrine (NE), and dopamine (DA) as potential factors in the etiology of major depressive disorder [52,53]. It has been found that eight active ingredients derived from *Bupleurum* can increase 5-HT levels and show significant antidepressant effects [54].

The monoamine hypothesis of depression continues to have proponents and detractors. While monoamine neurotransmitters likely play some role, depression is a heterogeneous and complex disorder. A more comprehensive understanding of the neurobiology of depression will require consideration of the interactions between monoamines and other neurotransmitter systems, neuroendocrine factors, circadian rhythms, and neuroplasticity [55].

### 2.5. Gut Microbiology

The precise environmental mechanisms underlying the pathophysiology of depressive disorder remain elusive. The gut microbiome is an increasingly recognized environmental factor that shapes the brain through the microbiota–gut–brain axis. Recent studies suggested that imbalanced gut microbiota may contribute to depression through host metabolism [56]. By restoring the relative abundance of intestinal flora and regulating the metabolic disorder of endogenous markers, the low polarity fraction of *Bupleurum radix* proved to be a potential treatment for depressive symptoms [57].

The pretreatment of mice with *Komagataella pastoris* prevented stress- and inflammation-induced depression-like behaviors in mice. It possesses the properties of a newly proposed probiotic with antidepressant-like effects and is a promising therapeutic strategy for depression [58]. Moreover, depression is linked to reduced gut microbiota diversity. Transplanting fecal microbiota from depressed patients into microbiota-depleted rats induces depression-like symptoms and changes in tryptophan metabolism [59].

**Figure 2:**
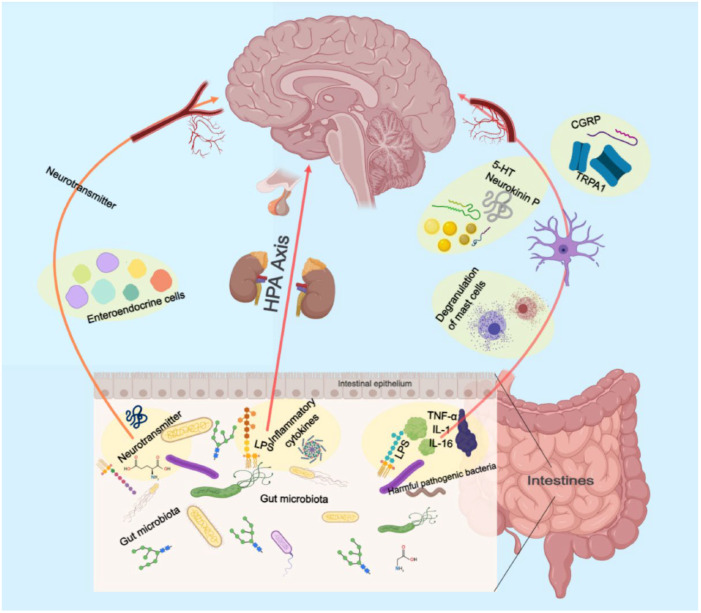
Schematic illustration of the role of the intestinal flora in gut–brain axis.

These findings suggest that the gut microbiome may play a vital role in depression (Figure 2). More research is needed to understand the mechanisms and develop novel treatments targeting the gut microbiome. 

## 3. Multiple Pharmacological Effects of *Bupleurum*

Phytochemical analysis of *Bupleurum* has revealed that it contains a variety of compounds, including saponins, polysaccharides, flavonoids, volatile oils, and others. These compounds have demonstrated significant pharmacological activities, such as antipyretic, anti-inflammatory, antioxidant, antitumor, improved glycolipid metabolism, and neuroprotective effects. Recent studies have highlighted the therapeutic potential of specific compounds found in *Bupleurum*. For example, Chai Hu aqueous extract had a mild but definite antipyretic effect on LPS-induced fever in rats by directly inhibiting the production of tumor necrosis factor alpha (TNF-α) by monocytes [60]. Another study showed that Saikosaponin A (SSA) had therapeutic potential in the treatment of oxidative liver injury through multiscale interactome-level analysis combined with experiments [61]. SSA also inhibited human bladder cancer T24 both in vitro and in vivo [62], while SSA extract had anticancer effects by inhibiting cell proliferation and inducing apoptosis in 5637 cells [63]. Saikosaponin D (SSD) could attenuate cancer cachexia by directly inhibiting signal transducer and activator of transcription 3 (STAT3) expression [64] as well as attenuate peripheral neuropathy in diabetic rats by regulating the Aquaporin 1 (AQP1)/Ras homolog family member A (RhoA)/Rho-associated protein kinase (ROCK) signaling pathway [65]. There is another report that Saikosaponins were able to ameliorate hyperlipidemia in rats by enhancing hepatic lipid and cholesterol metabolism [48]. Saikosaponin and Plumbagin had synergistic neuroprotective effects on corticosterone-induced apoptosis in PC12 cells by regulating metabolic disorders and neuroinflammation [66]. 

**Figure 3:**
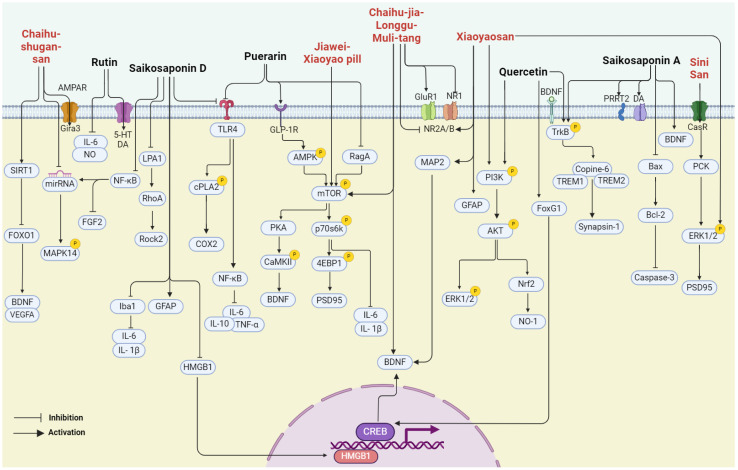
The multiple cellular targets of pharmacological components and related formulations of *bupleurum* with antidepressive effects.

In summary, *bupleurum* showed therapeutic potential for a wide range of health conditions through their antioxidant, anticancer, anti-inflammatory, and immunomodulatory effects. Unlike other natural medicines, *Bupleurum* is rich in a large number of saikosaponins. As a class of triterpene saponins, saikosaponins have been reported to have therapeutic potential in improving neuroplasticity [67,68,69]. However, the depressive effects of *bupleurum* have not been well summarized. In the following paragraph, we concluded that the major compounds isolated from *bupleurum* and the representative formula contain *bupleurum* with antidepressive effects (Figure 3) (Table 1). 

## 4. Pharmacological Components from *Bupleurum* with Antidepressive Effects

### 4.1. Rutin

Rutin, a common dietary flavonoid, is extensively ingested through plant-based foods and beverages, serving as a traditional and folk medicine across the globe. It is postulated that rutin possesses various pharmacological properties, encompassing antioxidant, anti-inflammatory, anti-diabetic, anti-adipogenic, neuroprotective, and hormonal therapeutic activities [97]. Studies have shown that rutin can effectively reduce the immobility time of depression-like rats in the forced swimming test and tail suspension test, showing an antidepressant effect. The antidepressant properties of rutin are attributed to elevated concentrations of NE, 5-HT, and DA in the cortical and hippocampal regions [70]. A recent investigation revealed that rutin reversed the decrease in sucrose preference in depression-like mice in the sucrose preference test. And its administration safeguarded against the loss of hippocampal neurons induced by chronic unpredictable stress, consequently yielding an antidepressant effect [71]. Additional research has suggested that the pretreatment of mice with the serotonin synthesis inhibitor PCPA effectively attenuated the decrease in immobility time induced by rutin. And the pretreatment of mice with AMPT, an inhibitor of tyrosine hydroxylase, significantly abolished the antidepressant-like effect of rutin in the tail suspension test. Rutin may manifest its antidepressant-like influences by augmenting the availability of serotonin and norepinephrine within the synaptic cleft [72]. The indoleaminergic pathway has been implicated in rutin’s preventive impact on alcohol-induced cognitive impairment and depression-like behavior in rat models [73].

### 4.2. Puerarin

Puerarin, a naturally occurring antioxidant, possesses considerable health-promoting properties. Its diverse range of biological activities encompasses antioxidative, anti-inflammatory, and anti-neoplastic properties, as well as immune system enhancement and the protection of cardiovascular, cerebrovascular, and neuronal cells [98]. Recent studies have indicated that puerarin treatment improved sucrose preference and depression-like behavior in HFD/CUMS-induced rats by inhibiting toll-like receptor 4 (TLR4)-mediated intestinal mucus barrier dysfunction and neuroinflammation damage through the TLR4/cytosolic phospholipases A2 (cPLA2)/cyclooxygenase-2 (COX-2) pathway [74]. Furthermore, puerarin exhibits antidepressant-like effects in HFD diabetic mice. It can increase the number of hippocampal neurons in HFD mice, inhibit neuronal apoptosis, and protect hippocampal neuroplasticity. Puerarin can enhance the expression of GLP-1R in the hippocampus of HFD mice, and then activate AMPK, CREB, and BDNF/TrkB signaling, thereby improving neuroplasticity [76]. Recent experimental findings suggest that puerarin can alleviate depression-like behavior in mice stimulated by LPS by inhibiting the Ras-related GTP-binding protein A (RagA), and it significantly reduced the expression of phosphorylated mTOR and phosphorylated p70S6K induced by LPS stimulation [77].

### 4.3. Quercetin

Quercetin is a naturally occurring flavonoid and ubiquitously present in various sources such as tea, coffee, apples, and onions. A plethora of research has demonstrated its multifaceted biological activities, including antioxidative, anti-inflammatory, and anti-aging properties [99]. The long-term administration of quercetin has been shown to significantly ameliorate CUMS-induced weight loss and depression-like behaviors in mice. Quercetin significantly reversed the protein levels of FoxG1, p-CREB, and BDNF in the hippocampus of mice compared with the CUMS-induced group [78]. Furthermore, Quercetin upregulated hippocampal Nrf2 and inhibited iNOS in CUMS-depressed mice, and it significantly reversed the decreased sucrose preference on the SPT [81]. Quercetin reduced the immobility time in the forced swimming test and tail suspension test in mice, which might be related to its ability to regulate the BDNF-related imbalance of Copine 6 and other synaptic-plasticity-related protein expressions in the hippocampus and prefrontal cortex. These results suggested that quercetin could improve LPS-induced depression-like behavior in rats [79]. In addition, Wang et al. showed that quercetin treatment significantly reduced the immobility time of TST and FST increases, significantly increased the protein expression level of BDNF in hippocampus and heart tissues of female mice, and increased the phosphorylation level of its downstream targets TrkB, AKT, and ERK1/2. The data suggested that quercetin exerts its antidepressant effect at least in part through BDNF and its downstream PI3K/AKT and MAPK/ERK intracellular signaling pathways [80]. 

### 4.4. Saikosaponin A

SSA is a triterpenoid saponin derived from Saikosaponin, exhibiting a broad spectrum of pharmacological properties [100]. Its antidepressant effects have been extensively documented. SSA has been shown to not only ameliorate depression-like behavior following cerebral ischemia through the cAMP-response element binding protein (CREB)/BDNF pathway and inhibit apoptosis of hippocampal neurons [35], but also alleviate perimenopausal depression-like symptoms. SSA could restore CUMS-induced hyperactivity of the HPA axis and pro-inflammatory cytokines as well as the downregulation of BDNF. SSA increased the expression of p-TrkB levels compared with model mice and promoted neuroplasticity in the hippocampus [82]. Furthermore, SSA may exert its antidepressant influence by upregulating the expression of proline-rich transmembrane protein 2 (PRRT2) and augmenting DA levels in the hippocampus. According to iTRAQ screening results, proteins, including PRRT2, SART1, CaMKII, DENND4B, DOHH, FMN1, TMEM160, REM2, SNX18, PTER, LIMD2, and GIMAP8, were downregulated during the CUMS procedure, while chronic treatment with SA (50 mg/kg daily) significantly counteracted this change [83]. 

### 4.5. Saikosaponin D

SSD is a biologically active triterpene saponin with anti-inflammatory, anticancer, antioxidant, and liver fibrosis inhibitory properties [101]. Research findings indicate that SSD exerts antidepressant effects by enhancing HPA axis function and promoting neurogenesis in the hippocampus [51]. In CUMS model rats, SSD mitigated the depression-like behavior by negatively regulating nuclear factor-kappa B (NF-κB), downregulating microRNA-155, and upregulating fibroblast growth factor 2 (FGF2) [84]. Additionally, its antidepressant effect has been linked to the regulation of Homer1-mGluR5 and mTOR signaling [102]. Further experimental results demonstrate that SSD alleviates depression-like behaviors and inhibits neuronal apoptosis by modulating the endogenous lysophosphatidic acid (LPA_1_)/RhoA/ROCK2 pathway in the LPS model, compared with the LPS group. SSD also significantly inhibited the activation of the MAPK/NfκB-p65 signaling [85]. Moreover, SSD attenuated LPS-induced depressive behaviors by inhibiting microglial activation and neuroinflammation, inhibited HMGB1 nuclear translocation, and decreased the protein levels of TLR4, p-inhibitor of nuclear factor kappa B-α (p-IκB-α), and NF-κB [86]. As for chronic mild stress (CMS), SSD has the potential to alleviate sexual behavior and neurological dysfunction, restore glial pathology, and suppress neuroinflammation and oxidative stress [68].

## 5. Representative Prescription

TCM, rooted in a rich history of thousands of years, offers a variety of herbal prescriptions and therapies for treating depressive disorders. There was a high occurrence of Chai Hu as an important Chinese herb in these antidepressant formulations. Some notable TCM prescriptions for depression include the following:

Chaihu-shugan-san (CSS) is a formulation comprising seven herb medicines, namely Chai Hu (*Bupleurum chinense* DC.), Xiang Fu (*Cyperus rotundus* L.), Zhi Qiao (*Citrus aurantium* L.), Chen Pi (*Citrus reticulata* Blanco), Chuan Xiong (*Ligusticum striatum* DC.), Bai Shao (*Paeonia lactiflora* Pall.), and Gan Cao (*Glycyrrhiza uralensis* Fisch). It is a prescription commonly used in traditional Chinese medicine. The research indicates that CSS can play an antidepressant role by changing intestinal flora and related metabolites, for example, by upregulating hyocholic acid (HCA), 7-ketodeoxycholic acid (7-ketoDCA), BDNF, and TrkB and downregulating farnesoid X receptor (Fxr) [87]. In addition, in another study, CSS promoted rat hippocampal synapse formation and MAPK14 mRNA and Gria3, and downregulated miR-503, miR-532, miR212, miR-125a, miR-182, and miR-124. Its effects are similar to but do not exceed fluoxetine [42]. CSS in the treatment of major depressive disease (MDD) also needs to have exact efficacy and safety guarantees. A study was conducted to screen whether CSS can induce angiogenesis and neurogenesis in the mouse hippocampus, increase SIRT1, reduce the expression of forkhead Box O1 (FOXO1), and upregulate vascular endothelial growth factor A (VEGFA) and BDNF, with the same result in brain microvascular endothelial cells (BMVECs) [88]. Deng et al. used CSS to evaluate its effect on cancer-related depression (CRD) in a large hospital in Beijing, and the results showed that CSS could effectively improve the anxiety and depression state of CRD patients [103].

Xiaoyao San (XYS) is one of the most famous prescriptions with a history of thousands of years. XYS is primarily used to treat liver stagnation and spleen deficiency syndrome. XYS is composed of Gan Cao (*Glycyrrhiza uralensis* Fisch.), Dang Gui (*Angelica sinensis* Diels.), Fu Lin (*Poria cocos* Wolf.), Bai Shao (*Paeonia lactiflora* Pall.), Bai Zhu (*Atractylodes macrocephala* Koidz.), and Chai Hu (*Bupleurum chinense* DC.). Clinical studies have shown that XYS can effectively improve the depression-like performance of patients, and after treatment, creatinine, taurine, 2-oxoglutarate in urine, and xanthine acid were significantly increased, whereas citrate, lactic acid, alanine, and dimethylamine were decreased [104]. The most recent study conducted by Jiao performed computational modeling on mice and concluded that XYS increases the expression of glutathione peroxidase 4 (GPX4), glial fibrillary acidic protein (GFAP), and ERK1/2, while it decreases acyl-coA synthetase long chain family member 4 (ACSL4), COX-2, total iron, ferrous content, and phosphatidylethanolamine-binding protein 1 (PEBP1). The opposite is true for p-ERK1/2 and ionized calcium-binding adapter molecule1 (Iba1) [90]. Their team also claimed that XYS reduced the levels of glutamate and serum corticosterone (CORT) in the hippocampus and enhanced microtubule-associated protein 2 (MAP2), N-methyl-D-aspartate receptor subunit 2B (NR2B), phosphatidylinositol-3-kinase (PI3K), and p-AKT, revealing that XYS may play an antidepressant role through NR2B and PI3K/Akt signaling pathways [91]. Another study showed that XYS improved depression-like behavior in rats by altering the gut flora. Specifically, at the phylum level, it modulates the abundance of different flora, and at the genus level, it decreases the abundance of *Prevotellaceae_Ga6A1* group, *Prevotellaceae_UCG-001*, and *Desulfovibrio*. The difference is that the abundance of *Ruminococcus* has been improved [92]. The results of XYS studied by senior researchers have shown that XYS can reverse the downregulation of BDNF caused by CUMS and promote the generation of Nestin-positive neurons and doublecortin-positive cells [89]. Wang et al. evaluated the efficacy of the XYS formulation in the treatment of post-stroke depression (PSD) in a clinical study involving 80 patients in Wuhan city. It is proved that it can effectively improve the degree of depression, neurological function, and activities of daily living in patients with mild PSD, improve the synthesis and release of neurotrophic factors, and has significant clinical efficacy and good safety evaluation [105].

The Jiawei-Xiaoyao pill (JWX) was recorded in the ancient Chinese official pharmacopoeia 900 years ago. JWX is composed of eight herbs: Chai Hu (*Bupleurum chinense* DC.), Dang Gui (*Angelica sinensis* Diels.), Bai Zhu (*Atractylodes macrocephala* Koidz.), Fu Ling (*Poria cocos* Wolf.), Gan Cao (*Glycyrrhiza uralensis* Fisch.), Mu Dan Pi (*Paeonia suffruticosa* Andr.), Zhi Zi (*Gardenia jasminoides* Ellis.), and Bo He (*Mentha haplocalyx* Briq.). Zhang et al. discovered that a single administration of JWX, within the prescribed dosage range, elicited rapid and sustained antidepressant effects in both normal mice and those with chronic depression. Furthermore, JWX swiftly enhanced neuroplasticity signaling and proteins such as mTOR, CaMKII, ERK, and BDNF in the hippocampus while also improving BDNF expression in the hippocampal dentate gyrus in mice models of depression induced by corticosterone exposure. They verified that activation of CaMKII is essential for stimulating the mTOR-BDNF signaling [93]. Wu et al. in a JWX control study involving 64 patients showed that JWX could improve the clinical symptoms and signs of patients with depression, improve the sleep quality of patients, and improve the quality of life of patients [106].

Sini San (SNS), another traditional Chinese herbal formula, is composed of four herbs: Chai Hu (*Bupleurum chinense* DC.), Bai Shao (*Paeonia lactiflora* Pall.), Zhi shi (*Citrus aurantium* L), and Gan cao (*Glycyrrhiza uralensis* Fisch.). SNS has antidepressant effects and improves synaptic plasticity through the calcium-sensitive receptor (CaSR)-protein kinase C (PKC)-ERK signaling pathway in rats. This study found that SNS treatment significantly upregulated the expression of CaSR, PKC, and p-ERK1/2 in the hippocampus and PFC of adult-stressed rats. This paper also reported that CaSR abnormalities are involved in the pathogenesis of depression [96]. In a clinical study of 100 patients with postpartum depression, Wang et al. found that SNS could improve the clinical symptoms of patients and relieve anxiety and depression, interpersonal communication skills, and quality of life [107].

Chaihu-jia-longgu-muli tang (CLM) is a classic Chinese herbal medicine used in the treatment of depression. CLM is composed of 11 Chinese herbs, including Chai Hu (*Bupleurum chinense* DC.), Long Gu (longgu), Huang Qin (*Scutellaria baicalensis* Georgi.), Sheng Jiang (*Zingiber officinale* Rose), Ren Shen (*Panax ginseng* C. A. Mey), Gui Zhi (*Cinnamomum cassia* Presl), Fu Ling (*Poria cocos* Wolf.), Ban Xia (*Pineilia ternata* Breit.), Da Huang (*Rheum palmatum* L.), Mu Li (*Ostrea gigas* Thunberg.), and Da Zao (*Ziziphus jujuba* Mill.). The researchers found that CLM produced immediate and lasting antidepressant activity by enhancing hippocampal BDNF expression, in which components of the Xiaochuhu decoction played a major role [94]. In addition, CLM induced rapid antidepressive-like effects in OB mice, which may be due to the reversal of abnormal expression of AMPA and NMDA receptors in the PFC and the related Akt-mTOR signaling [95]. In a clinical observation involving 121 patients, Tang et al. found that CLM had a certain clinical efficacy in the treatment of patients with mild to moderate essential hypertension complicated with depression, which could improve depression and reduce inflammatory response while reducing blood pressure [108].

## 6. Summary and Perspective

*Bupleurum*, a traditional herbal medicine, is widely used in Asian countries to treat ailments. With advancements in modern pharmacology, analytical chemistry, and other disciplines, researchers have identified many compounds in *Bupleurum*, including volatile oils, triterpenoid saponins, flavonoids, lignans, fatty acids, and sterols. Among these, triterpene saponins, flavonoids, and volatile oils exhibit various pharmacological activities and are considered the main active ingredients of *Bupleurum* [109]. Several compounds, such as saikosaponin A, quercetin, and rutin, possess antidepressant properties. These active ingredients are effective in treating depression by regulating neurotransmitters (e.g., 5-HT, DA), brain-derived neurotrophic factor (BDNF), and their intracellular signaling pathways. They also protect neurons through anti-inflammatory and antioxidant activities and regulate the expression of related proteins. 

At present, there are still many limitations in the study of the antidepressant effect of *bupleurum*. The current literature reports are mostly based on animal or cell models, and the relationship between the target and the molecular mechanism needs to be further studied. Many radix bupleurum formulas, such as CSS, XYS, and SNS, are frequently used in clinical applications in China and Japan, but the specific active ingredients of radix bupleurum are still in the experimental stage. There is a lack of randomized double-blind testing of TCM compounds, and stronger evidence-based medicine is needed to prove the clinical role of bupleurum. 

In addition, concerns about bupleurum toxicity are commonly reported, and it is believed that the long-term use of bupleurum can cause abnormal liver function [110,111]. It has been described that high doses (>19 g) of bupleurum increase the risk of liver failure in patients with hepatitis B [112]. This is much higher than the dose used in clinical practice and Chinese pharmacopoeia prescribes a daily dose of 3 to 10 g. In 2016, the Chinese Association of Chinese Medicine issued the clinical diagnosis and treatment guidelines for TCM-related liver injury to standardize the use of traditional Chinese herbs [113]. The use of bupleurum in regular medical institutions, in accordance with the guidance of the pharmacopoeia, is deemed safe.

As a natural product, *Bupleurum* contains a rich variety of pharmacological components and antidepressant active ingredients. Several compounds mentioned in this paper, such as saikosaponin A in *Bupleurum*, are about 0.35–0.63%. The content of saikosaponin C was about 0.15–0.27%, and the content of saikosaponin D was about 0.35–0.9%. The content of puerarin in *Bupleurum* was 0.04–0.145 mg/g [16,114], the content of rutin was 1.533–19.88 mg/g, and the content of quercetin was 0.059–0.927 mg/g [115]. And they provide a new avenue for scientific research: investigating whether two or more antidepressant active ingredients can produce a synergistic effect. For example, our previous study found that the co-administration of geniposide and shanzhiside methyl ester was able to increase hippocampal PACAP production, thereby synergizing to produce rapid antidepressant effects [116]. In this article, we only discussed the mechanism of action of single antidepressant active constituents of *Bupleurum*. The potential for synergistic antidepressant effects among compounds isolated from *Bupleurum* warrants further investigation in future studies. The prevalence of depression is rising yearly. Given the depth of fundamental research on depression, the translation of scientific findings into practice and the strengthening of clinical research and treatment of this condition should be actively promoted. 

**Figure 4:**
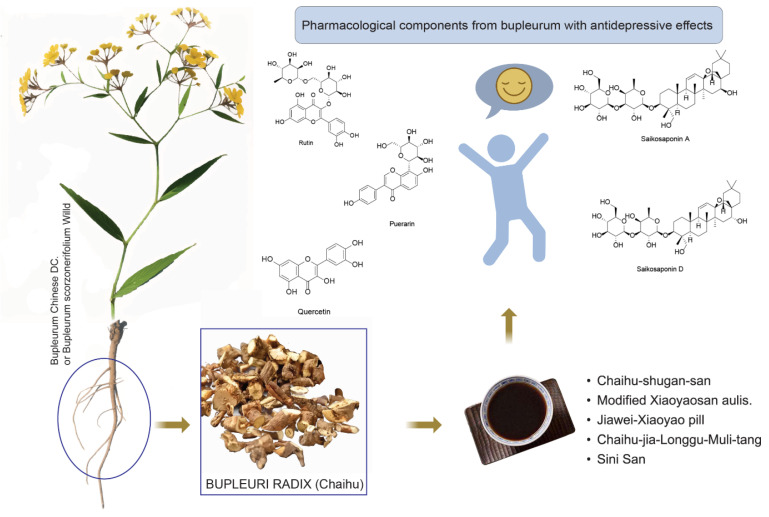
Graphical abstract of Chai Hu.

This article primarily introduces *Bupleurum* and its chemical constituents, focusing on recent advancements regarding its antidepressant active ingredients and the potential antidepressant mechanisms of relevant TCM prescriptions. It provides a theoretical foundation and clinical application for the treatment of depression using TCM (Figure 4).

## Figures and Tables

**Table 1:** The overview of underlying mechanisms of potential compounds and present prescriptions from Chai Hu with antidepressive effects.

Drug	Chemical Structrue	Animal Species	Model	Route of Administration	Dosage	Administration Time	Antidepressant Mechanism	Reference
Rutin	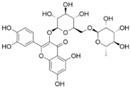	The Male/feMale Sprague–Dawley rats	Reserpine	i.p.	40 mg/kg 80 mg/kg	/	serotonin ↑, norepinephrine ↑, dopamine levels ↑	[70]
		Adult Swiss albino mice	CUS	Oral	100 mg/kg	3 weeks	/	[71]
		Male Swiss mice	PCPA, AMPT	Oral	0.3 mg/kg	60 min	serotonin and norepinephrine in the synaptic cleft ↑	[72]
		Adult Male Wistar rats	Ethanol	Oral	50 mg/kg	5 weeks	IDO ↑; antioxidant enzymes ↓; NO, IL-6, and MPO ↓	[73]
Puerarin	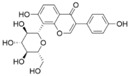	Male Sprague–Dawley (SD) rats	HFD, CUS	/	30 mg/kg 60 mg/kg 120 mg/kg	1 week	IL-6, TNF-α, and IL-10 ↓; occludin and claudin-1 ↑; TLR4 ↓, cPLA2, and COX-2 ↓; PGE 2 ↓	[74]
		Male ICR mice	CUMS	Oral Gavage	30 mg/kg 100 mg/kg	4 weeks	The abundance of pathogenic bacteria ↓, the abundance of beneficial bacteria ↑	[75]
		Male C57BL/6 mice HT22cell	HFD db/db	Oral gavage	150 mg/kg	6 weeks	AMPK ↑, AKT ↑, mTOR ↓, BDNF ↑, TrkB ↑, CREB ↑, ERK ↑, GLP-1R ↑, 5-hydroxytryptamine ↑, serum levels of corticosterone and IL-1β ↓	[76]
		Male C57BL/6N mice PC12cell	LPS	Gavage	30 mg/kg 60 mg/kg 120 mg/kg	/	RagA ↓, P-mTOR and P-70S6K ↓, LAMP2 ↑, IL-6 and IL- 1β ↓	[77]
Quercetin	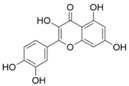	Male ICR mice SH-SY5Y Cell	CUMS	Gavage	15, 30 mg/kg 60 μM	3 weeks	NSC ↑, AHN ↑, FoxG1 ↑, p-CREB ↑, BDNF ↑	[78]
		Male Sprague–Dawley rats	LPS	Gavage	40 mg/kg	15 days	BDNF, p-TrkB/TrkB, Copine 6, and TREM1 ↑; TREM2 ↑; Synapsin-1 ↑	[79]
		ERα-KO mice in C57BL/6 J b	ERα -KO	Oral administration	100 mg/kg	10 weeks	Number of hippocampal neurons ↑; Bcl-2 ↑; BDNF, P-TrkB, P-AKT, and p- ERK1/2 ↑	[80]
		Adult Male Kunming mice	CUMS	Gavage	10, 20, 40 mg/kg	3 weeks	P-PI3K ↑, P-Akt ↑, Nrf2 ↑, HO-1 ↑, iNOS ↑, NO ↑, MDA ↑, T-SOD ↑, GST ↑, T-SOD ↑	[81]
Saikosaponin A	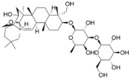	Adult Male Sprague–Dawley (SD) rats	MCAO + isolation + CUMS model	i.p.	5 mg/kg	once daily for 24 days	p-CREB ↑, BDNF ↑, Bcl-2 ↑, Bax ↓, Caspase-3 ↓	[35]
		FeMale Wistar rats	CUMS	Oral	25 mg/kg, 50 mg/kg, 100 mg/kg	4 weeks	the serum corticosterone levels ↑, CRH ↑, IL-1β ↓, IL-6 ↓, TNF-α ↓, BDNF ↑, P-TrkB/TrkB ↑	[82]
		Forty-five Male Sprague–Dawley (SD) rats	CUMS	Gavage	50 mg/kg	4 weeks	PRRT2 ↑, DA ↑	[83]
Saikosaponin D	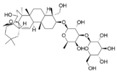	Sprague– Dawley (SD) rats	CUMS	Gavage	0.75 mg/kg, 1.50 mg/kg	3 weeks	NF-kB, miR-155 ↓, FGF2 ↑	[84]
		Male ICR mice	LPS	Gavage	0.5 mg/kg, 1 mg/kg	2 weeks	MAPK/NFκB-p65 ↓	[85]
		Male ICR mice	LPS	Gavage	1 mg/kg	7 days	HMGB1 nuclear translocation ↓; TLR4/NF-kB, p-IkB-α ↓	[86]
		C57BL/6 J	CMS	Gavage	1 mg/kg	3 weeks	GFAP ↑, Iba1 ↓, IL-1β, IL-6, ROS ↓	[68]
Chaihu-shugan-san	/	Male C57BL/6 J mice	CUMS	gavage	20 mg/kg/d	8 weeks	BA ↑, BDNF ↑, TrkB ↑, Fxr ↓, HCA ↑, 7-ketoDCA ↑	[87]
		Male Sprague–Dawley rats	CUMS	Gavage	2.835 g/kg	4 weeks	Synapse formation in the hippocampus ↑, miR-503 ↓, miR532 ↓, miR212 ↓, miR-125a ↓, miR-182↓, miR-124 ↓, MAPK14 ↑, Gria3 ↑	[42]
		Male C57BL/6 mice, Male Sprague–Dawley rats	CUMS	Gavage	19.5 g herb/kg	6 weeks	SIRT1 ↑, FOXO1 ↓, VEGFA ↑, BDNF ↑	[88]
Xiaoyaosan aulis.	/	Male C57BL/6 J mice	CUMS	Gavage	0.4 g/kg	6 weeks	BDNF ↑, Nestin-positive neurons and DCX-positive cells ↑	[89]
		/	CUMS	Gavage	0.254 g/kg	3 weeks	GPX4 ↑, FTH1 ↓, ACSL4 ↓, COX2 ↓, total iron ↓, ferrous content ↓, PEBP1 ↓, t-ERK1/2 ↑, p-ERK1/2 ↑, p-ERK1/2 to t-ERK1/2 ↓, GFAP ↑, IBA1 ↓	[90]
		Male Sprague–Dawley rats	CUMS	Gavage	2.224 g/kg	3 weeks	glutamate ↓, CORT ↓, MAP2 ↑, NR2B ↑, PI3K ↑, P-AKT/AKT ↑	[91]
		Male Sprague–Dawley rats	CRS	Gavage	2.224 g/kg	3 weeks	Bacteroidetes, Proteobacteria, *Firmicutes*, Chloroflexi, and *Planctomycetes* ↑; *Prevotellaceae_Ga6A1*_group, *Prevotellaceae_UCG-001*, and *Desulfovibrio* ↓; *Ruminococcaceae* family ↑	[92]
Jiawei-Xiaoyao pill	/	Male ICR mice	The mouse corticosterone (CORT) model for depression	Gavage	0.7, 1, 1.4, 1.8 g/kg	single dose	pmTOR/mTOR ↑, pCaMKII/CaMKII ↑, pERK/ERK ↑, BDNF ↑, PSD95 ↑ and synapsin1 ↑	[93]
Chaihu-jia-Longgu-Muli-tang	/	BALB/c mice	-	Gavage	4.2 g/kg	single dosage	BDNF ↑	[94]
		Kunming mice	OB	Gavage	2.1 g/kg	single dosage	GluR1 ↑, NR1 ↓, NR2A ↓, NR2B ↓, total Akt ↑, pAKT ↑, total mTOR ↑, P-mTOR ↑, pmTOR/mTOR ↑	[95]
Sini San	/	Male Sprague–Dawley rats	CUMS	Gavage	2.5 g/kg, 5 g/kg, 10 g/kg	40 days	PSD-95 ↑, GAP-43 ↑, Syn ↑, CaSR ↑, p-ERK _1/2_ ↑, PKC ↑	[96]

## Data Availability

No new data were created or analyzed in this study. Data sharing is not applicable to this article.

## Abbreviations

7-ketodeoxycholic acid: 7-ketoDCA; acyl-coA synthetase long chain family member 4: ACSL4; Aquaporin 1: AQP1; brain microvascular endothelial cells: BMVECs; brain-derived neurotrophic factor: BDNF; calcium-sensitive receptor: CaSR; cAMP-response element binding protein: CREB; Chaihu-shugan-san: CSS; chronic mild stress: CMS; chronic unpredictable mild stress: CUMS; cognitive behavioral therapy: CBT; corticosterone: CORT; cyclooxygenase-2: COX-2; cytosolic phospholipases A2: cPLA2; dopamine: DA; electroconvulsive therapy: ECT; endogenous lysophosphatidic acid: LPA1; extracellular signal-regulated kinase1/2: ERK1/2; farnesoid X receptor: Fxr; fibroblast growth factor 2: FGF2; Forkhead Box O1: FOXO1; genome-wide association study: GWAS; glucagon-like peptide-1 receptor: GLP-1R; high-fat diet: HFD; hyocholic acid: HCA; hypothalamus–pituitary–adrenal: HPA; Interleukin-6: IL-6; interpersonal psychotherapy: IPT; ionized calcium-binding adapter molecule1: Iba1; Kynurenine: Kyn; lipopolysaccharide: LPS; major depressive disease: MDD; mammalian target of rapamycin: mTOR; microtubule-associated protein 2: MAP2; modified Xiaoyao San: MXYS; Nerve Growth Factor-Inducible protein A: NFGI-A; Nod-like receptor protein 2: NLPR2; norepinephrine: NE; nuclear factor-E2-related factor 2: NFE2L2; Nuclear factor-kappa B: NF-κB; nucleus accumbens: Nac; p70 ribosomal S6 kinase: p70S6K; phosphatidylethanolamine-binding protein 1: PEBP1; phosphatidylinositol-3-kinase: PI3K; p-inhibitor of nuclear factor kappa B-α: p-IκB-α; prefrontal cortex: PFC; proline-rich transmembrane protein 2: PRRT2; protein kinase B: AKT; protein kinase C: PKC; Ras homolog family member A: RhoA; Ras-related GTP-binding protein A: RagA; repetitive transcranial magnetic stimulation: rTMS; Rho-associated protein kinase: ROCK; Saikosaponin A: SSA; Saikosaponin D: SSD; serotonin: 5-HT; signal transducer and activator of transcription 3: STAT3; Sini San: SNS; toll-like receptor 4: TLR4; traditional Chinese medicine: TCM; triggering receptor expressed on myeloid cells-1/2: TREM1/2; Tropomyosin receptor kinase B: TrkB; tumor necrosis factor alpha: TNF-α; vagus nerve stimulation: VNS; vascular endothelial growth factor A: VEGFA; Xiaoyao San: XYS.
